# Effects of Machining Errors on Optical Performance of Optical Aspheric Components in Ultra-Precision Diamond Turning

**DOI:** 10.3390/mi11030331

**Published:** 2020-03-23

**Authors:** Yingchun Li, Yaoyao Zhang, Jieqiong Lin, Allen Yi, Xiaoqin Zhou

**Affiliations:** 1 School of Mechatronic Engineering, Changchun University of Technology, Changchun 130012, China; chun04230525@126.com (Y.L.); 2201801055@stu.ccut.edu.cn (Y.Z.); 2 Department of Industrial, Welding and systems Engineering, Ohio State University, Columbus, OH 43210, USA; 3 School of Mechanical and Aerospace Engineering, Jilin University, Changchun 130022, China

**Keywords:** optical aspheric surface, fast tool serve (FTS), machining error, optical performance

## Abstract

Optical aspheric components are inevitably affected by various disturbances during their precision machining, which reduces the actual machining accuracy and affects the optical performance of components. In this paper, based on the theory of multi-body system, we established a machining error model for optical aspheric surface machined by fast tool servo turning and analyzed the effect of the geometric errors on the machining accuracy of optical aspheric surface. We used the method of ray tracing to analyze the effect of the surface form distortion caused by the machining error on the optical performance, and identified the main machining errors according to the optical performance. Finally, the aspheric surface was successfully applied to the design of optical lens components for an aerial camera. Our research has a certain guiding significance for the identification and compensation of machining errors of optical components.

## 1. Introduction

As a new type of optical surface, an optical aspheric surface has obvious advantages, including correcting aberration, reducing the size and weight of the system, expanding the field of view, compared with a traditional regular surface, and has become a core component of modern optical systems [1,2,3]. In order to meet the actual optical performance of the components, it is necessary to rely on high-precision machining. However, the processing of components will inevitably be affected by various factors, such as geometric error, tool error, and thermal error [4,5]. All error components are reflected on the surface of the workpiece through the kinematic chain of the machine tool, which causes the form distortion of actual machined surface and affects optical performance of the components [6]. Therefore, in order to improve the machining accuracy of aspheric components, it is necessary to perform an error analysis on the machining process.

Usually, the geometric error is a basic factor influencing the machining accuracy [7]. Leete [8], based on trigonometric relationship, established a geometric error model for a three-axis computer numerical control (CNC) machine tools. Based on the assumption of rigid body motion and small angle error, Ferreira [9] proposed an analytical model for the prediction of geometric error of a three-axis machine tool. Okafor [10] used homogeneous transformation matrix (HTM) to model and analyze the geometric error and thermal error of the vertical three-axis machining center. Lamikiz et al. [11] used Denavit–Hartenberg (D–H) convention to model the geometric error of the machining center. Based on the theory of multi-body system (MBS), Kong et al. [12] established a volumetric error model for ultra-precision machine tools. In addition, some scholars use neural network theory and stream of variation theory to analyze machine tool errors [13,14].

Due to the existence of geometric error of machine tool, the form distortion will occur on the surface of the workpiece being machined. The sensitivity of the geometric errors’ effect on form error is different for machining surface, so the compensation of the errors with high sensitivity is more effective [15]. Li et al. [16] used the matrix differential method to study the effect of geometric error on machining accuracy. Cheng et al. [17] identified main geometric errors for a multi-axis machine tool based on the MBS theory. Note that in previous studies, the evaluation of machining quality of components only relies on geometric form. However, as an applied device, the processing of optical aspheric components should meet the needs of optical applications [18].

The main evaluation parameters of optical performance for optical surfaces include wavefront aberration [19,20], modulation transfer function (MTF) [21], point spread function (PSF) [22] and power spectral density (PSD) [23,24], etc. As a comprehensive index, wavefront aberration can transform with other evaluation parameters [18], so the evaluation parameter based on wavefront aberration was applied in this paper to study the influence of machining errors.

In this paper, based on the mothed of creating optical aspheric components by FTS turning, we studied the geometric error modeling of the machine tool, the modeling of three-dimensional topography for machined surface, and the evaluation modeling of optical performance. The aim of this paper was to establish the relationship between the geometric error of machine tool and the form error of machined surface and the optical performance of aspheric surface, and to realize the identification of main machining errors based on optical performance, so as to guide the machining for optical aspheric components with specific optical performance and promote the wide application of aspheric surface.

## 2. Volumetric Error Modeling

**Figure 1:**
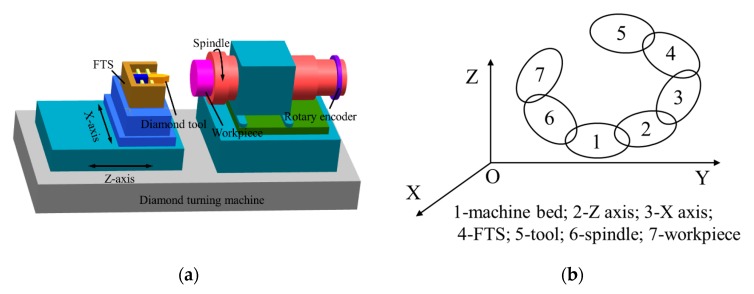
(**a**) Schematic diagram and (**b**) Kinematic chain diagram of the fast tool serve (FTS) turning system.

It is important for the analysis, separation and compensation of the machining errors to establish the volumetric error model of the machine tool [25,26]. The schematic diagram of FTS turning system is shown in Figure 1a. In the process of machining for aspheric surfaces, the tool reciprocates linearly in the axial direction under the driving of the FTS, which can be viewed as a prismatic pair along Z-direction. Therefore, the system includes three translational axes and one rotational axis, i.e., X axis, Z axis, FTS axis and C axis, respectively.

The number of error components depending on the number of axes of a particular machine. Since each axis of the machine system has six degrees of freedom, it has six error components. From the geometric errors analysis, it can be seen that the FTS turning system has 24 error components provided by X-axis, Z-axis, C-axis and FTS-axis and 6 squareness errors. However, in order to simplify the error model, only the squareness error between X-axis and Z-axis were considered among the 6 squareness errors. Table 1 shows the 26 geometric errors of the FTS turning system.

### 2.1. Transformation Matrix between Adjacent Bodies

The relative location and attitude between two bodies can be obtained through HTM based on the MBS theory [27]. The topology of the FTS turning system is shown in Figure 1b, which includes a tool branch form the machine bed to the cutting tool and a workpiece branch from the machine bed to the workpiece.

When machining aspheric surfaces with FTS turning, the Z-axis carriage of machine tool is only used to calibrate the initial position of the X-axis carriage and does not participate in the machining motion. Therefore, the connection between the Z-axis carriage and the machine bed be regarded as static, that the HTM $T_{12}=I_{4\times 4}\;$. Moreover, there is no relative movement between the tool and FTS, that is $T_{4}^{5}=I_{4\times 4}\;$.

The misalignments between the X-axis carriage and the Z-axis carriage cause a small offset called the squareness error $\eta_{zx}\;$. Therefore, the HTM $T_{2}^{3}$ between the X-axis carriage and the Z-axis carriage should take into account the effect of the squareness error $\eta_{zx}\;$.

(1) $$T_{2}^{3}=\left[\begin{array}{ccc} 1 & -\varepsilon_{xz} & \varepsilon_{xy}\\ \begin{array}{c} \varepsilon_{xz}\\ -\varepsilon_{xy}\\ 0 \end{array} & \begin{array}{c} 1\\ \varepsilon_{xx}\\ 0 \end{array} & \begin{array}{c} -\varepsilon_{xx}\\ 1\\ 0 \end{array} \end{array}\;\begin{array}{c} R-x+\delta_{xx}\\ \begin{array}{c} \delta_{xy}\\ \delta_{xz}-x\eta_{zx}\\ 1 \end{array} \end{array}\right]$$

where R represents the radius of the workpiece being machined and x represents the amount of movement of the X-axis carriage during processing.

The FTS device is mounted on the X-axis carriage, and the tool holder coupled to it moves in the Z-direction respect to the X-axis carriage. So the HTM $T_{3}^{4}$ from the X-axis carriage to the FTS can be formulated as follows after considering the error components listed in Table 1.

(2) $$T_{3}^{4}=[\begin{array}{cccc} 1 & -\varepsilon_{f_{z}} & \varepsilon_{fy} & \delta_{fx}\\ \varepsilon_{f_{z}} & 1 & -\varepsilon_{fx} & \delta_{fy}\\ -\varepsilon_{fy} & \varepsilon_{fx} & 1 & S-z_{f}+\delta_{fz}\\ 0 & 0 & 0 & 1 \end{array}]$$

where S represents the surface Sag value of the optical aspheric component being machined; $z_{f}$ represents the amount of movement of the tool holder in the Z direction during the processing.

In practice, the actual rotation center of the spindle will shift from the nominal rotation center. When the spindle rotates θ angle relative to the bed, the HTM $T_{1}^{6}$ between them can be represented as Equation (3) with the assumption of small angle approximation.

(3) $$T_{1}^{6}=[\begin{array}{cccc} cos\theta -\varepsilon_{cz}sin\theta & -sin\theta -\varepsilon_{cz}cos\theta & \varepsilon_{cy}cos\theta +\varepsilon_{cx}sin\theta & \delta_{cx}cos\theta -\delta_{cy}sin\theta \\ sin\theta +\varepsilon_{cz}cos\theta & cos\theta -\varepsilon_{cz}sin\theta & \varepsilon_{cy}sin\theta -\varepsilon_{cx}cos\theta & \delta_{cy}cos\theta +\delta_{cx}sin\theta \\ -\varepsilon_{cy} & \varepsilon_{cx} & 1 & \delta_{cz}\\ 0 & 0 & 0 & 1 \end{array}]$$

Since the workpiece is fixed on the spindle, the HTM $T_{6}^{7}$ from the workpiece to the spindle is also a unitary matrix, that is, $T_{6}^{7}=I_{4\times 4}\;$.

### 2.2. Integrated Volumetric Errors Model

The process of developing the integrated errors model mainly aims to obtain the relative displacement error between the cutting tool and the workpiece in the turning process [28]. Assume that the cutting tool tip position in tool coordinate system (TCS) is as follows.

(4) $$P_{t}={\left[x_{t},\;y_{t},\;z_{t},\;1\right]}^{T}$$

Then, the cutting tool position in the workpiece coordinate system (WCS) can be formulated as

(5) Pw=(T17)−1T15Pt

where

(6) T17=T16T67

(7) T15=T12T23T34T45

Under ideal conditions, all the errors in Table 1 are equal to zero. Combining the Equations (6) and (7), the ideal form-shaping function (the ideal cutting tool position in the WCS) can be expressed as Equation (8):

(8) Pw0=(T170)−1(T150)Pt=[cosθ−sinθ00sinθcosθ0000100001]−1[100R−x010000100001][10000100001S−Zf0001][xtytzt1]

In the actual machining process, the cutting tool point is the combination of the ideal cutting tool point and the error motion [29]. Therefore, considering the geometric error terms of machine tool in Table 1, the actual shape-forming function (the actual cutting tool position in the WCS) is formulated as Equation (9):

(9) Pw=(T17)−1(T15)Pt=([cosθ−sinθ00sinθcosθ0000100001][1−εczεcyδcxεcz1−εcxδcy−εcyεcx1δcz0001])−1=([100R−x010000100001][1−εxzεxyδxxεxz1−εxxδxy−εxyεxx1δxz+xηzx0001][10000100001S−Zf0001][1−εfzεfyδfxεfz1−εfxδfy−εfyεfx1δfz0001])(xtytzt1)

Combining Equations (8) and (9), the volumetric error E of FTS turning system can be obtained.

(10) $$E={\left[E_{x},\;E_{y},\;E_{z},\;1\right]}^{T}=P_{w}-P_{w0}$$

Finally, the position deviation of the cutting tool in the WCS can be expressed as follows (ignore the higher-order terms):

(11) $$\begin{array}{ll} E_{x}= & \left(\delta_{fx}+\delta_{xx}\right)cos\theta +\left(\delta_{fy}+\delta_{xy}\right)sin\theta -\varepsilon_{xx}\left(S-z_{f}\right)sin\theta \\ & +\varepsilon_{xy}\left(S-z_{f}\right)cos\theta +\varepsilon_{cz}\left(x-R\right)sin\theta -\delta_{cx}-\varepsilon_{cy}\left(S-z_{f}\right)\\ E_{y}= & \left(-\delta_{fx}-\delta_{xx}\right)sin\theta +\left(\delta_{fy}+\delta_{xy}\right)cos\theta -\varepsilon_{xx}\left(S-z_{f}\right)cos\theta \\ & -\varepsilon_{xy}\left(S-z_{f}\right)sin\theta +\varepsilon_{cz}\left(x-R\right)cos\theta -\delta_{cy}+\varepsilon_{cx}\left(S-z_{f}\right)\\ E_{z}= & \left(\delta_{xz}+\delta_{fz}-\delta_{cz}\right)+\varepsilon_{cx}\left(R-x\right)sin\theta +\varepsilon_{cy}\left(R-x\right)cos\theta -H_{zx}x \end{array}$$

## 3. The Influence of Machining Errors

According to the effect of the error components on the coordinate distortions [29], the error components in Equation (11) are simplified to 11 errors as shown in Table 2. The influence of 11 simplified errors on the coordinate distortions is different.

These main error components should be considered in the machining and compensation process. Therefore, 12 sets of simulation cases are constructed to study the influence of geometric error components in the process of machining aspheric surface with FTS turning. Due to the distribution trend of the coordinate distortions will not change with the change of error values, the position errors of machine tool are generally less than 0.001mm, and the angle errors are generally less than 0.001°, so all error values are set to 0.001mm ($0.001^{{}^{\circ}}$) for simulation. The simulated plans are listed in Table 3.

### 3.1. Effect on Form Distortion

Due to the existence of the coordinate distortions in the X, Y and Z directions caused by the machining errors, there is a form deviation between the actual (considering the error terms in Table 1) surface and ideal (each error terms in Table 1 is 0) surface. According to the plans in Table 3, simulation is performed using a toric surface to study the effect of machining error components on the form distortion of optical aspheric surface. The toric surface is expressed as Equation (12).

(12) $$z=R_{b}-\sqrt{{\left(R_{b}-R_{d}+\sqrt{R_{d}^{2}-\rho^{2}sin^{2}\theta }\right)}^{2}-\rho^{2}cos^{2}\theta }$$

where $R_{b}=265\;mm$ and $R_{d}=132.5\;mm$ are set in this article. 

**Figure 2:**
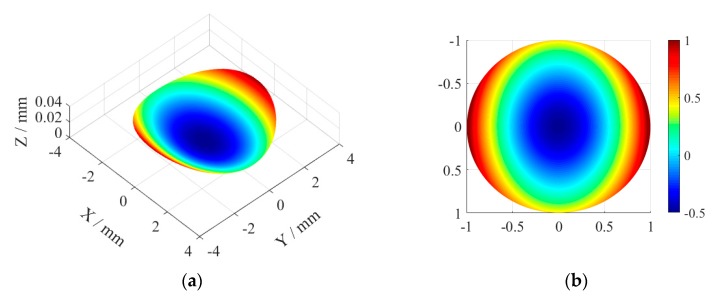
Under ideal conditions, toric surface’s (**a**) Three-dimensional topography map of the machined surface and (**b**) Wavefront map.

In the simulation, the tool arc radius is set as r=0.5 mm, the spindle speed is N=500 r/mm and the feed rate is set as $a_{f}=0.02\;\mathrm{mm}/r$. Under ideal conditions, the turning surface topography is shown in Figure 2a. The rotary asymmetry and bisymmetry characteristics of the toric surface can be seen clearly, which is the desired surface under the Cartesian coordinate system.

Under the actual machining situation, the existence of geometric errors of machining system will affect the shape accuracy of the machined surface for components, and the influence of different geometric errors is also different. The main error components should be given priority in the processing and compensation of components. Figure 2a shows the contribution of the machining error terms to the form distortion by calculating the root mean square deviation ($S_{q}$) values of the form errors. Among them, the form distortion data is obtained from the surface topography by wavelet analysis, which only contains the low-frequency components.

**Figure 3:**
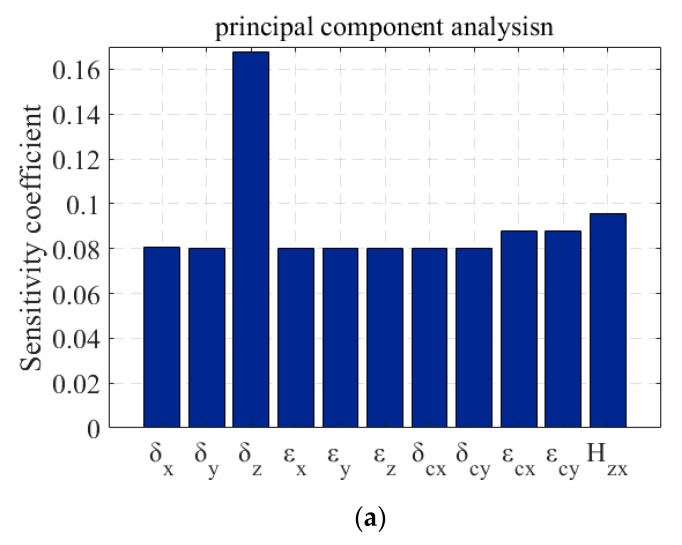

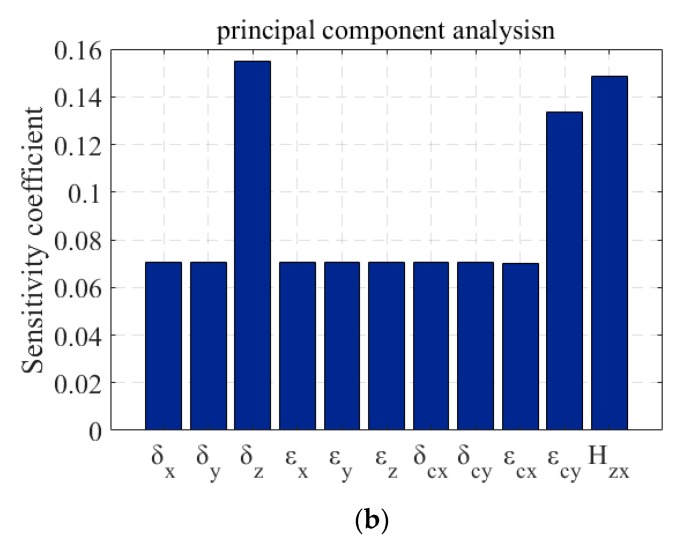
The contribution of different error components: (**a**) To form distortion; (**b**) To wavefront increment.

Principal component analysis can be used to find the main error terms [28]. As can be from Figure 3a, the contribution of error $\delta_{z}$ is significantly larger than that of other errors in the figure, which is the main machining error term affecting the form accuracy.

### 3.2. Effect on Optical Performance

The form distortion of optical aspheric components will affect their own optical performance. Wavefront aberration is the optical path difference between the actual wavefront and the ideal wavefront, form which one can easily derive MTF, PSF, and other optical parameters [18,30]. In this paper, the wavefront aberration was obtained by using the Zernike polynomials to fit the discrete data with the same phase after the light is refracted (or reflected).

Figure 2b shows the wavefront map corresponding to the machined toric surface under the ideal conditions. Under the actual machining situation, for aspheric surface, the form distortions caused by different machining error components are different, and different form distortions will also cause different aberrations. It has been proven that although the form distortions of the two surfaces had similar peak to valley (PV) values, their final optical performances were obviously different due to the different distribution of the errors [26]. Therefore, the principal component analysis of machining errors based on optical performance is performed in this paper.

The increment of wavefront aberration is the deviation of the wavefront under the condition with error disturbances from that the wavefront under the ideal machining condition. As shown in Figure 3b, the contribution of the geometric error components to the wavefront increment by calculating the $S_{q}$ values of the wavefront aberration increment. It can be seen from the figure that the contributions of error $H_{\mathrm{zx}}$ and error $\varepsilon_{\mathrm{cy}}$ to wavefront increment are close to that of error $\delta_{z}\;$, which are all the main machining errors that affect the optical performance of the components. In combination with Figure 3a, it can be concluded that the form distortions caused by the error $H_{\mathrm{zx}}$ and the error $\varepsilon_{\mathrm{cy}}$ will greatly reduce the optical performance of the components compared with the form distortions caused by other errors. Error $H_{zx}\;$, $\varepsilon_{cy}\;$, and $\delta_{z}$ are all the main machining error terms of toric surface created with FTS turning.

### 3.3. Details of the Effect on Optical Performace

In this paper, since the wavefront was obtained by fitting using Zernike polynomials and Zernike polynomials correspond to the primary aberrations, wavefront aberration is a comprehensive reflection of multiple geometric aberrations. The size of Zernike fitting coefficients symbolizes the size of different geometric aberrations. From Figure 3, it can be seen that the error components $\delta_{z}\;$, $H_{\mathrm{zx}}$ and $\varepsilon_{\mathrm{cy}}$ are the main machining error terms in the process of creating toric surface with FTS turning. Therefore, the first nine items of the fitting coefficients of the wave surface under ideal condition and the wave surface under cases C, J and K are listed in the Table 4 to further analyze the effect of machining errors on optical performance.

It can be seen from Table 4 that due to the influence of machining errors, the fitting coefficients of the wavefront of the actual toric surface is larger than the ideal coefficients, that is, in the actual machining process of the optical aspheric components, the existence of the geometric errors will increase the wavefront aberration and the primary aberrations of the components and reduce the optical performance of the optical components. In addition, by comparing the values of fitting coefficients of Zernike polynomials under different conditions, it can be concluded that the variation of coefficients $q_{5}$ and $q_{6}$ is larger than that of other coefficients, which correspond to the focus and Y-astigmatism, that is, the machining errors has the greatest impact on these two aberrations.

**Figure 4:**
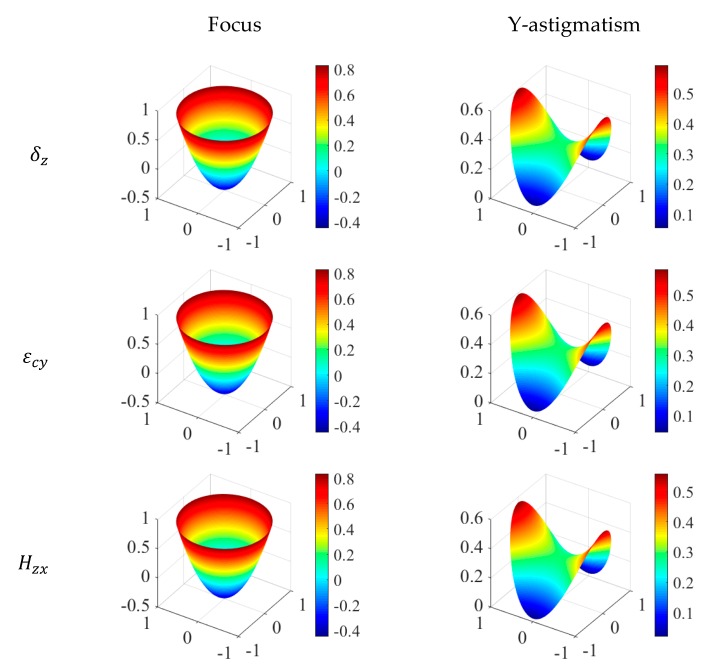
The focus map and the Y-astigmatism map under the influence of errors $\delta_{z}\;$, $\varepsilon_{cy}$ and $H_{zx}$.

**Figure 5:**
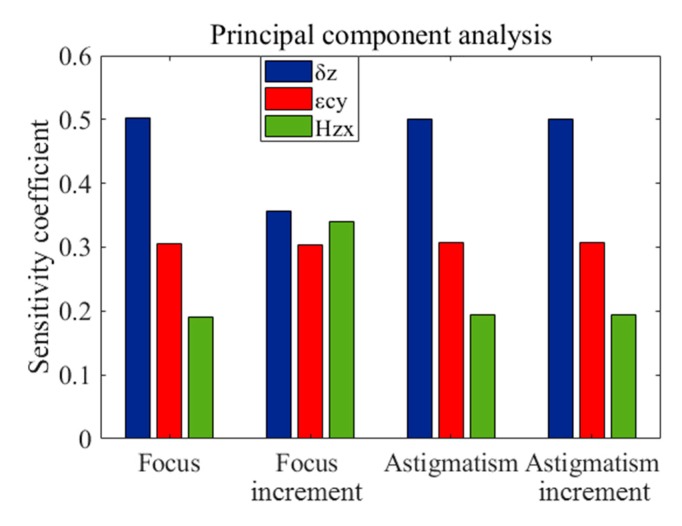
The contribution of three main error components $\delta_{z}\;$, $\varepsilon_{cy}$ and $H_{zx}$.

In order to further clarify the details of the influence on geometric aberration, a principal component analysis was carried out to identify the main machining errors that affect the focus and the Y-astigmatism of the optical aspheric surface. As shown in Figure 4, the maps of focus and Y-astigmatism under the influence of three main machining errors of $\delta_{z}\;$, $H_{\mathrm{zx}}$ and $\varepsilon_{\mathrm{cy}}\;$, and Figure 5 is the analysis of main machining errors affecting the two aberrations.

The following conclusions can be drawn from Figure 4 and Figure 5.

(1) The machining error $\delta_{z}$ is not only the main contributor to the wavefront aberration, but also the main contributor to focus and Y-astigmatism;

(2) According to the contribution to focus, the three machining errors can be sorted, $\delta_{z}>\varepsilon_{cy}>H_{zx}\;$, but they have the same impact on the focus increment.

(3) The contributions of the three main machining errors to the Y-astigmatism and the increment of Y-astigmatism all can be sorted, $\delta_{z}>\varepsilon_{cy}>H_{zx}\;$.

(4) In the process of machining aspheric surface with FTS turning, we should first control and compensate the geometric error component $\delta_{z}$ ($\delta_{xz}\;$, Z-direction displacement error of X-axis; $\delta_{fz}\;$, Z-direction displacement error of FTS-axis; $\delta_{cz}\;$, Z-direction displacement error of spindle) of the lathe, and then $\varepsilon_{cy}$ (Y-direction angular error of spindle) and $H_{zx}$ (the squareness error between the X and Z axis).

## 4. Compensation for Main Machining Errors

The form quality and optical performance of the machined surface mainly depend on the main machining errors. In Section 3, the main error components in the actual machining process of optical aspheric surface with FTS turning are $\delta_{z}\;$, $H_{\mathrm{zx}}$ and $\varepsilon_{\mathrm{cy}}\;$. Therefore, in order to improve the quality of machined surface, the main machining error components are compensated in this section.

**Figure 6:**
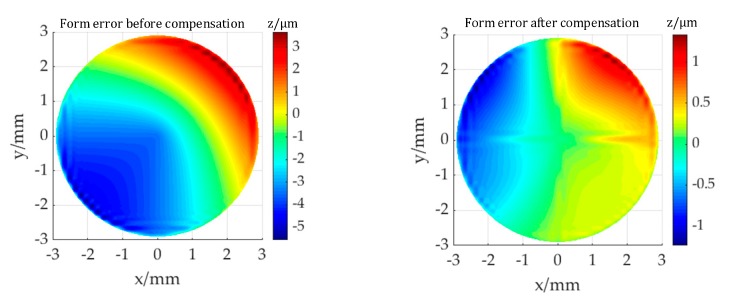
Form error of the machined toric surface: (**a**) before compensation; (**b**) after compensation.

In Equation (11), the position deviation of the cutting tool under the influence of the errors is shown. It can be seen that the three main geometric error components mainly affect the Z coordinate, and distortion values is also obtained. Error compensation requires that a value equal to the distortion be superimposed in the direction opposite to the distortion direction, i.e., $E_{z}\;$. The identified machining errors were compensated by modifying the tool path to improve the form equality and the optical performance in this section. As shown in Figure 6a, the form error before compensation for the machined toric surface, with a $S_{q}$ value of 9.25 μm. The form error after compensation, with a $S_{q}$ value of 2.26 μm, is shown in Figure 6b. Compared to the form accuracy before compensation, the accuracy is improved by 82.29%.

Zernike polynomial coefficient corresponding to wavefront aberration before and after compensation is shown in Table 5, and the coefficient after compensation is reduced. This reduction in form error and Zernike coefficients proves the validity and effectiveness of improving optical performance by only compensation the distortion of z-coordinate for cutting tool.

## 5. Conclusions

Based on the theory of machining optical aspheric surface by FTS turning, the volumetric error modeling and the influence of machining errors are studied in this paper. The effect of the geometric errors on the form accuracy and optical performance is analyzed and simulated, which can make us understand the machining errors effect law in nature. The main conclusions that can be drawn are as follows:

(1) The error components $\delta_{z}\;$, $\varepsilon_{cy}$ and $H_{zx}\;$, as the main machining errors, have an impact on the form accuracy and optical performance of the optical aspheric components, and the contribution of the error $\delta_{z}$ is the largest. 

(2) The influence of three main machining error components on wavefront aberration is mainly through increasing the focus and the Y-astigmatism of the optical aspheric surface.

(3) The three main error components affect the form accuracy of machined surface mainly by causing z-coordinate distortion of cutting tool. In the actual process of machining with FTS turning, the compensation in z coordinate of cutting tool will play an active in the form quality and optical performance of the machined components.

## Figures and Tables

**Table 1:** Geometric error components of the fast tool serve (FTS) turning system.

Axis	Error Terms
Axis X	$\delta_{xx},\;\delta_{xy},\;\delta_{xz},\;\varepsilon_{xx},\;\varepsilon_{xy},\;\varepsilon_{xz}\;$
Axis Z	$\;\delta_{zx},\;\delta_{zy},\;\delta_{zz},\;\varepsilon_{zx},\;\varepsilon_{zy},\;\varepsilon_{zz}\;$
Spindle (Axis C)	$\;\delta_{cx},\;\delta_{cy},\;\delta_{cz},\;\varepsilon_{cx},\;\varepsilon_{cy},\;\varepsilon_{cz}\;$
Axis FTS	$\;\delta_{fx},\;\delta_{fy},\;\delta_{fz},\;\varepsilon_{fx},\;\varepsilon_{fy},\;\varepsilon_{fz}\;$
Squareness error	$\;\eta_{zx}\;$

$\delta_{mn}$: Displacement errors; $\varepsilon_{mn}$: Angular error, where the first subscript refers to the motion axis, the second subscript refers to the error direction or the rotation axis of angular error, $\eta_{zx}$: the squareness error between axis X and axis Z.

**Table 2:** The simplified model of the machining errors.

Error Terms	Coordinate Distortion in the X Direction *E_x_*	Coordinate Distortion in the Y Direction *E_y_*	Coordinate Distortion in the Z Direction *E_z_*
$\;\delta_{x}=\delta_{fx}+\delta_{xx}\;$	$\;\delta_{x}\cos\theta \;$	$-\delta_{x}sin\theta \;$	0
$\;\delta_{y}=\delta_{fy}+\delta_{xy}\;$	$\;\delta_{y}sin\theta \;$	$\;\delta_{y}cos\theta \;$	0
$\;\delta_{z}=\delta_{xz}+\delta_{fz}-\delta_{cz}\;$	0	0	$\;\delta_{z}\;$
$\;\varepsilon_{xx}\;$	$-\varepsilon_{x}\left(S-z_{f}\right)sin\theta \;$	$-\varepsilon_{x}\left(S-z_{f}\right)cos\theta \;$	0
$\varepsilon_{xy}\;$	$\varepsilon_{y}\left(S-z_{f}\right)cos\theta \;$	$-\varepsilon_{y}\left(S-z_{f}\right)sin\theta \;$	0
$\;\varepsilon_{cz}\;$	$\;\varepsilon_{z}\left(x-R\right)sin\theta \;$	$\;\varepsilon_{z}\left(x-R\right)cos\theta \;$	0
$\;\delta_{cx}\;$	$-\delta_{cx}\;$	0	0
$\;\delta_{cy}\;$	0	$-\delta_{cy}\;$	0
$\;\varepsilon_{cx}\;$	0	$\;\varepsilon_{cx}\left(S-z_{f}\right)\;$	$\;\varepsilon_{cx}\left(R-x\right)sin\theta \;$
$\;\varepsilon_{cy}\;$	$-\varepsilon_{cy}\left(S-z_{f}\right)\;$	0	$\;\varepsilon_{cy}\left(R-x\right)cos\theta \;$
$\;H_{zx}\;$	0	0	$-H_{zx}x\;$

**Table 3:** Simulation plans and error values.

Case No.	*δ_x_* (mm)	*δ_y_* (mm)	*δ_z_* (mm)	*ε_xx_* (mm)	*ε_xy_* (mm)	*ε_cz_* (mm)	*δ_cx_* (mm)	*δ_cy_* (mm)	*ε_cx_* (mm)	*ε_cy_* (mm)	*H_zx_* (mm)
A	0.001	0	0	0	0	0	0	0	0	0	0
B	0	0.001	0	0	0	0	0	0	0	0	0
C	0	0	0.001	0	0	0	0	0	0	0	0
D	0	0	0	0.001	0	0	0	0	0	0	0
E	0	0	0	0	0.001	0	0	0	0	0	0
F	0	0	0	0	0	0.001	0	0	0	0	0
G	0	0	0	0	0	0	0.001	0	0	0	0
H	0	0	0	0	0	0	0	0.001	0	0	0
I	0	0	0	0	0	0	0	0	0.001	0	0
J	0	0	0	0	0	0	0	0	0	0.001	0
K	0	0	0	0	0	0	0	0	0	0	0.001
L	0.001	0.001	0.001	0.001	0.001	0.001	0.001	0.001	0.001	0.001	0.001

**Table 4:** Zernike fitting coefficients of the wavefront of machined toric surface under different conditions.

Coefficient No. *q_i_*	Ideal Coefficients	Actual Coefficients under the Influence of Different Errors		
		*δ_z_*	*ε_cy_*	*H_zx_*
$\;q_{1}\;$	0.017775607	0.177403762	0.17765288	0.180462849
$\;q_{2}\;$	2.60 × 10^-18^	3.10 × 10^−7^	3.32 × 10^−7^	2.74 × 10^−7^
$\;q_{3}\;$	−6.59 × 10^−5^	−0.002374094	−0.00244473	−0.002048713
$\;q_{4}\;$	4.22 × 10^−18^	2.86 × 10^−7^	3.06 × 10^−7^	2.53 × 10^−7^
$\;q_{5}\;$	0.311282774	0.34964832	0.35004824	0.3513772
$\;q_{6}\;$	−0.148267872	−0.128465135	−0.128461443	−0.128424573
$\;q_{7}\;$	4.33 × 10^−18^	−1.35 × 10^−7^	−1.38 × 10^−7^	−1.21 × 10^−7^
$\;q_{8}\;$	9.10 × 10^−18^	2.51 × 10^−7^	2.41 × 10^−7^	2.25 × 10^−7^
$\;q_{9}\;$	−1.28 × 10^−5^	−0.003751194	−0.003833412	−0.003912768

**Table 5:** Zernike coefficients for wavefront aberration before and after Z-coordinate distortion compensation.

Zernike Item	Before Compensation	After Compensation
$q_{5}$	0.356139841	0.355403992
$q_{6}$	−0.127976253	−0.127969512
